# Aluminum-Alumina Composites: Part I: Obtaining and Characterization of Powders

**DOI:** 10.3390/ma12193180

**Published:** 2019-09-27

**Authors:** Alexander A. Gromov, Anton Yu. Nalivaiko, Grayr N. Ambaryan, Mikhail S. Vlaskin, Olesya A. Buryakovskaya, Sergey A. Kislenko, Andrey Z. Zhuk, Evgeniy I. Shkolnikov, Konstantin V. Slyusarskiy, Alexandra A. Osipenkova, Alexey N. Arnautov

**Affiliations:** 1 KINETICA Engineering Center, National University of Science and Technology MISIS, 119991 Moscow, Russia; a.gromov@misis.ru (A.A.G.); slyuskonst@gmail.com (K.V.S.); 79859100168@ya.ru (A.A.O.); aleksej.arnautov@gmail.com (A.N.A.); 2 Laboratory of Energy Storage Substances, Joint Institute for High Temperatures of the Russian Academy of Sciences, 125412 Moscow, Russia; ambaryan1991@gmail.com (G.N.A.); vlaskin@inbox.ru (M.S.V.); osminojishe@ya.ru (O.A.B.); kislenko-s@mail.ru (S.A.K.); 666zhuk@ihed.ras.ru (A.Z.Z.); 2shkolnikov@ihed.ras.ru (E.I.S.)

**Keywords:** aluminum, alumina, composite, powder, oxidation, selective laser melting, particle morphology

## Abstract

The process of advanced aluminum-alumina powders production for selective laser melting was studied. The economically effective method of obtaining aluminum-alumina powdery composites for further selective laser melting was comprehensively studied. The aluminum powders with 10–20 wt. % alumina content were obtained by oxidation of aluminum in water. Aluminum oxidation was carried out at ≤200 °C. The oxidized powders were further dried at 120 °C and calcined at 600 °C. Four oxidation modes with different process temperatures (120–200 °C) and pressures (0.15–1.80 MPa) were investigated. Parameters of aluminum powders oxidation to obtain composites with 10.0, 14.5, 17.4, and 20.0 wt. % alumina have been determined. The alumina content, particle morphology, and particle size distribution for the obtained aluminum-alumina powdery composites were studied by XRD, SEM, laser diffraction, and volumetric methods. According to the obtained characteristics of aluminum-alumina powdery composites, they are suitable for the SLM process.

## 1. Introduction

Selective laser melting (SLM) for 3D metal object production is a rapidly developing field of science and technology. 3D printing of aluminum alloys and aluminum matrix composites (AMC) is pretending to become the leading technology for the production of complex shape details for aerospace and automotive engineering [1,2,3,4,5,6,7].

The SLM process parameters (laser power, scanning speed, powder feeding rate, etc.) have a decisive influence on the 3D object characteristics [8,9,10,11]. The quality of the initial powder is quite significant as well [12,13]. The properties of aluminum-based alloys are well suited to produce complex shape objects of high strength and density by SLM processes [14].

There are many problems in structure formation (for example, porosity) of 3D sintered objects formed by using the powders with a broad particle size distribution. The powders with spherical particles having a narrow size distribution is the best initial material to obtain 3D objects with high quality by SLM. This provides a compact packaging of particles in a melted layer and a stable feeding rate of the powder. For example, aluminum powders with a particle size from 30 to 100 μm are typically used for 3D printing by the SLM process [15,16].

Disadvantages of aluminum powders for additive technologies are their poor flowability, low emissivity factor, high conductivity, and low mechanical properties of synthesized 3D objects [17,18]. On the opposite side, aluminum powders reinforced with refractory additives allow to obtain sintered objects with excellent properties, such as good wear resistance, high hardness, and tensile strength [19]. In such powders, aluminum is the matrix phase and it forms a percolating network during sintering, and the refractory additive is the reinforcement and crystallization center [20]. SiC, TiC, TiB_2_, or Al_2_O_3_ can be used as a refractory additive [21]. However, the use of Al_2_O_3_ eliminates the possibility of formation of side phases during sintering and interaction of the reinforcement with the aluminum matrix. aluminum-alumina composites are considered as raw materials for the synthesis of potential lightweight and high-strength alloys for aircraft and automotive industry [19].

Aluminum particles covered with an alumina shell (Al core and Al_2_O_3_ shell) is an interesting raw material for 3D printing. The Al core and Al_2_O_3_ shell structure of the particles has higher stability, emissivity, thermal resistance, and aging properties in comparison with non-oxidized aluminum particle [22]. The flowability of such powder is also much higher due to lower surface energy and, as a result, lesser cohesion forces. Despite some advantages, high aluminum oxide content in the obtained object could decrease its strength properties. That is why alumina content in composites should be strictly controlled.

**Figure 1:**
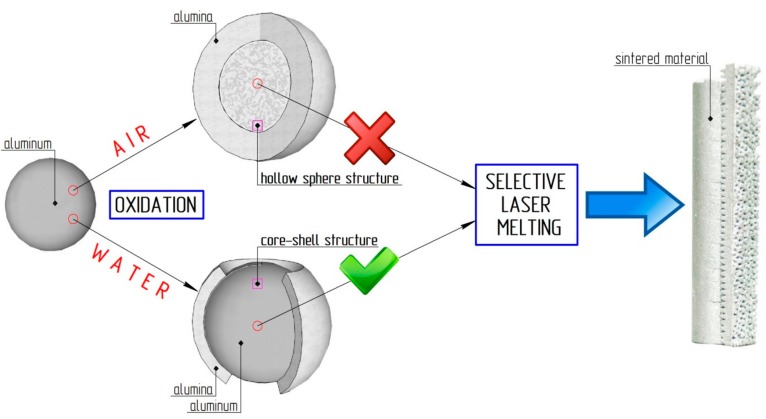
Scheme of the slow oxidation of aluminum particle by air and water.

There are different ways of obtaining aluminum-alumina powder composites from aluminum powder. The most obvious is the oxidation of aluminum by air or oxygen [23,24,25]. However, the oxidation of small particles causes the formation of hollow alumina spheres [23]. As the initial material for the SLM process, hollow alumina has unacceptable characteristics (see Figure 1). Oxidation of aluminum powder in water is one of the possible solutions [26,27]. The aluminum particle oxidation process can be stopped at any oxidation degree by reagent separation (water and aluminum) at a certain time. The oxidized powder can be deleted from the water by filtering or sedimentation. This method is perfectly suitable for producing Al core and Al_2_O_3_ shell powdery composites.

The oxidation of nano- and micron-sized aluminum powders by water is widely studied [22,23,26,27,28,29,30,31,32,33]. The majority of publications [22,28,29,30,31,34] dealt with the different points of obtaining hydrogen by the reaction of aluminum with water. The primary purpose of such works is an increase in the Al + H_2_O reaction rate because aluminum is passivated by oxide layer [26,31,34]. Many works on the aluminum powder oxidation by water and steam are devoted to the theoretical aspects of the aluminum oxidation and hydrogen obtaining [28,34]. Some studies have shown that high oxidation temperatures lead to the following problems: Poorly ordered crystal structure of oxidation products, agglomeration of particles, low specific surface area and micropores in oxidation products [31]. The effect of mechanochemical activation on the reaction kinetics in Al–H_2_O system was studied, and the effect of alkali metals was investigated [22,28,29,30]. The oxidation of the micron-sized aluminum powder by water for the synthesis of pore-free aluminum-alumina composites was also proposed [35]. This oxidation leads to the formation of aluminum particles with an oxide surface layer (Al core and Al_2_O_3_ shell) [28,31,36].

The aim of this work is to study the process of oxidation of aluminum with water for the manufacture of Al core and Al_2_O_3_ shell powder composites and the obtained powder’s comprehensive characterization.

## 2. Materials and Methods

### 2.1. Aluminum Powder

Micron-sized aluminum powder (purchased from RUSAL Co., Russia) was used as the initial material to obtain aluminum-alumina composite. Aluminum powder was produced by molten aluminum spraying. The elemental composition of aluminum powder was studied by using the ThermoScientific X–2 mass spectrometer with inductively coupled plasma gun (ICP–MS method). The powder size distribution curves were obtained with the Fritsch Analysette 22 particle size analyzer.

### 2.2. Aluminum-Alumina Powder Composites

The method of obtaining the aluminum-alumina composite powder consisted of two stages:

1. Oxidation of aluminum powder by water at pressure 0.15–1.80 MPa;
2. Powder drying at 120 °C and calcination of the aluminum-alumina composite in a furnace at 600 °C.

#### 2.2.1. Oxidation of Aluminum Powder by Water (Stage 1)

The first stage of obtaining the aluminum-alumina powder composites was the oxidation of aluminum powder by water. The oxidation process was executed in the high-pressure reactor which was described in [37]. Three kilograms of suspension of aluminum powder and distilled water (mass ratio of H_2_O/Al = 2) was added into the reactor. The reactor had a volume of five liters, and it was made of stainless steel with Teflon thermal isolation inside. An anchor-type agitator with 100 rotations per minute stirred the suspension continuously.

The temperature and the pressure inside the reactor were controlled by thermocouple type K (Ni–CrNi) and pressure sensor. When the pressure and temperature values were above a certain limit, additional cool water was added to the reactor to reduce its temperature. High heating is associated with the exothermic reaction of aluminum oxidation, which emits from 418 to 438 kJ/(mol Al) of heat (Equations (1) and (2))

Al + 3 H_2_O → Al(OH)_3_ + 1.5 H_2_ − 438 kJ (1)

Al + 2 H_2_O → AlOOH + 1.5 H_2_ − 418 kJ (2)

The H_2_O/Al suspension was poured into the reactor, and the suspension was continuously mixed. The suspension was heated at 3 K/min rate to a certain temperature (see Table 1). The mixture of hydrogen and steam was transferred to the heat exchanger where the water was condensed and returned to the reactor. Hydrogen was a byproduct, and it was released into the atmosphere. Solid products of the reaction (Al + Al(OH)_3_ + AlOOH) were removed from the bottom of the reactor. The resulting solid reaction products (Al + Al(OH)_3_ + AlOOH) were separated from the residual water by the filtration process.

#### 2.2.2. Drying and Calcination (Stage 2)

The thermal treatment consisted of drying and calcination.

The wet powder from Stage 1 (Al + Al(OH)_3_ + AlOOH) was loaded into stainless steel containers. The container was placed in the Binder VD drying box. The drying process was executed for 1 h at 120 ° C.

Then, the dried powder was subjected to the calcination. This stage was necessary for the conversion of all crystalline modifications of oxidation products (Al(OH)_3_, AlOOH, etc.) to γ–Al_2_O_3_. The calcination process was carried out for five hours at 600 °C (LHT 08/16 Nabertherm laboratory furnace).

#### 2.2.3. Characterization

Products of chemical interaction of powdery aluminum with water (reactions (1) and (2)) were identified in [28]. Reactions (1) and (2) were proceeded in parallel depending on the process temperature. According to [22], oxidation products after Stage 1 were presented by Al(OH)3, AlOOH, and θ-Al_2_O_3_. The possibility of γ–Al_2_O_3_ formation from reactions 1 and 2 was not reported. Further thermal treatment was needed to convert aluminum hydroxides into the aluminum oxides (3).

AlOOH/Al(OH)_3_ → Al_2_O_3_ + H_2_O, T = 600 °C (3)

The alumina content in the resulting aluminum-alumina powdery composites was determined by the amount of hydrogen, which was released during the oxidation of aluminum powder by water. However, aluminum-alumina composites were further oxidized during the calcination process. Therefore, the aluminum content in the finally obtained composites was determined by the volumetric method [25].

The morphological properties of the finally obtained aluminum-alumina composites were studied on the JEOL JSM–7407F microscope. The phase composition of the composites was studied by the EQUINOX 1000 X-ray diffractometer.

## 3. Results

### 3.1. Aluminum Powder Characterization

**Figure 2:**
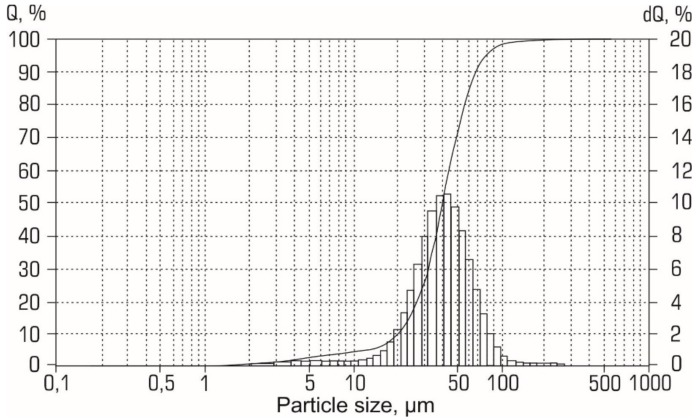
Size distribution curve for the initial aluminum powder.

The elemental composition of the initial aluminum powder is presented in Table 2. The most significant impurities were Ga (0.09 wt. %), Zn and Ce (both 0.08 wt. %), Fe, and La (both 0.07 wt. %). According to the size distribution curves (see Figure 2), the particles of the initial Al powder had a diameter from 1 to 120 µm.

**Figure 3:**
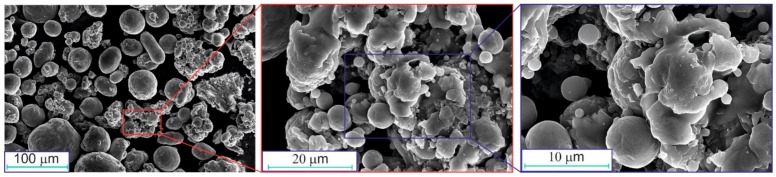
SEM images of the initial aluminum powder.

SEM images of the initial aluminum powder are shown in Figure 3. The particles were spherical or spheroidal. Several agglomerates consisting of smaller spherical particles were observed.

Thus, the low impurities content together with the relatively narrow particle size distribution and spherical particles’ shape makes this initial aluminum powder suitable as a raw material for further oxidation and core–shell composites preparation.

### 3.2. Oxidation of Aluminum Powder

**Figure 4:**
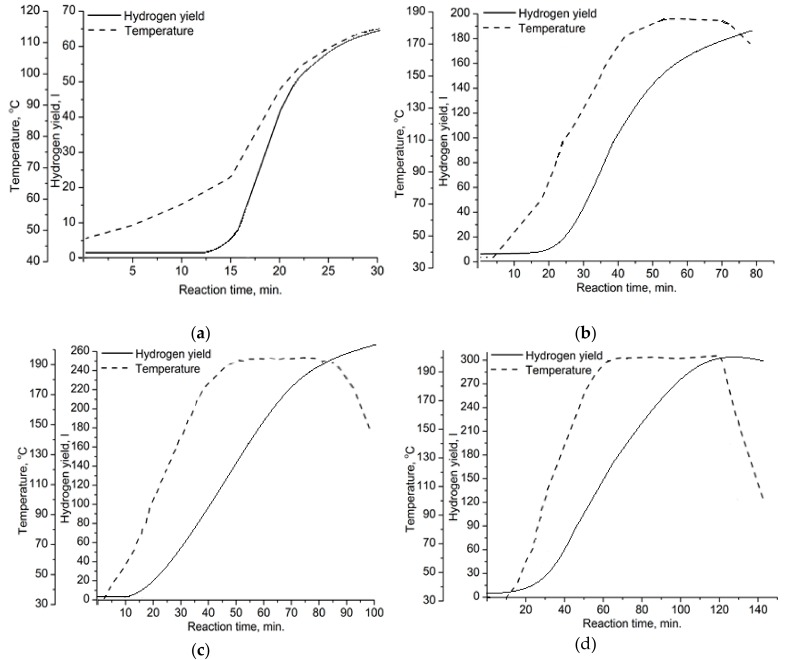
Yield of the released hydrogen and reaction temperature vs. reaction time for the following oxidation modes: (**a**) A, (**b**) B, (**c**) C, (**d**) D.

Four oxidation modes were studied to obtain aluminum-alumina powdery composites with the alumina content from 10.0 wt. % for mode A (see Figure 4a) to 20.0 wt. % for mode D (see Figure 4d and Table 1). The higher amount of released hydrogen for mode D compared to mode A corresponded to a higher alumina content in the powder. The higher alumina content correlated with the higher oxidation temperature and longer reaction time (see Figure 4). According to Figure 4, oxidation of aluminum by water was initiated at a temperature of about 68 °C. This can be explained by the partial permeability of the surface oxide layer at this temperature.

The curves of hydrogen yield (Figure 4) had approximately the same slope for all studied oxidation modes. The oxidation rate was increased linearly as the oxidation temperature increased. The reason for that was the active self-sustaining exothermic reaction of aluminum with water, which has been reported in numerous publications [22,30,31,34]. For the studied modes, the suppression of the reaction rate due to the protective layer formation on aluminum particles was not found. 

The alumina content in composites directly depended on the oxidation duration: For mode A the oxidation duration was 30 min and the alumina content was 10 wt.%. For mode D, the oxidation duration was 140 min and the alumina content was 20 wt.%.

### 3.3. Effect of the Oxidation Mode on the Particles’ Properties

**Figure 5:**
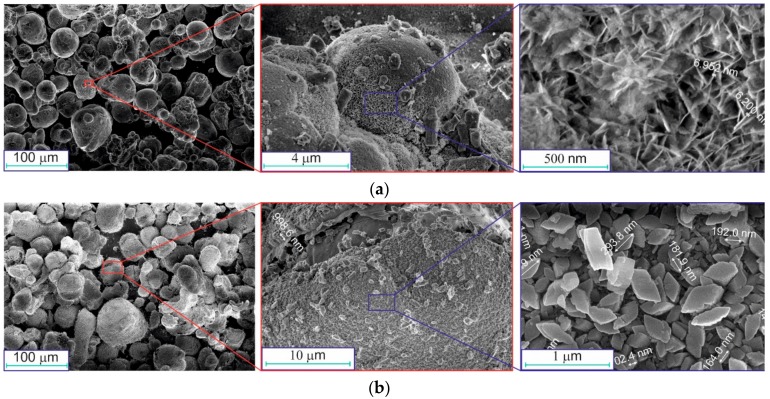
SEM images of the oxidized aluminum powders at different modes (before drying and calcination): (**a**) A, (**b**) D.

SEM images of the obtained aluminum-alumina powdery composites (before drying and calcination) are shown in Figure 5.

Comparison of the images in Figure 3 and Figure 5 shows that aluminum particles did not change their spherical shape after oxidation in water. The relatively smooth surface of aluminum particles was covered with a gibbsite-boehmite layer of oxidation products (see Figure 5a, right image). The hydroxide layer on the particles had an irregular structure formed by plate crystals (Figure 5a). Agglomerates of particles were also observed. The size of these agglomerates was larger than 100 µm (see Figure 5b, left image) and they were larger than the average diameter of Al particles (see Figure 3, left image).

After deep oxidation (mode D), the aluminum particles did not change their shape (see Figure 5b). A small part of the hydroxide layer was separated from the surface, and individual particles were present in the sample (see Figure 5b, right image). The hydroxide layer on the particles oxidized in mode D consists of larger crystals, and it looks denser.

**Figure 6:**
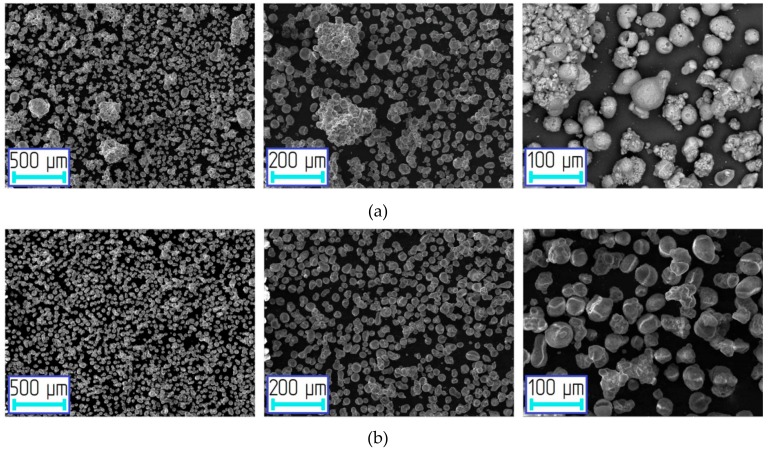
SEM images of the oxidized aluminum powders at different modes (after drying and calcination processes): (**a**) A, (**b**) D.

SEM images of aluminum-alumina powdery composites after heat treatment are shown in Figure 6. The particles were predominantly spherical. Nevertheless, there were some agglomerates, which are explained by the sintering of particles during the calcination process. After the deep oxidation (mode D) there were no agglomerates (see Figure 6b). A thicker oxide surface layer was preventing the sintering of particles. The presence of agglomerates had a little effect on the particle size distribution.

**Figure 7:**
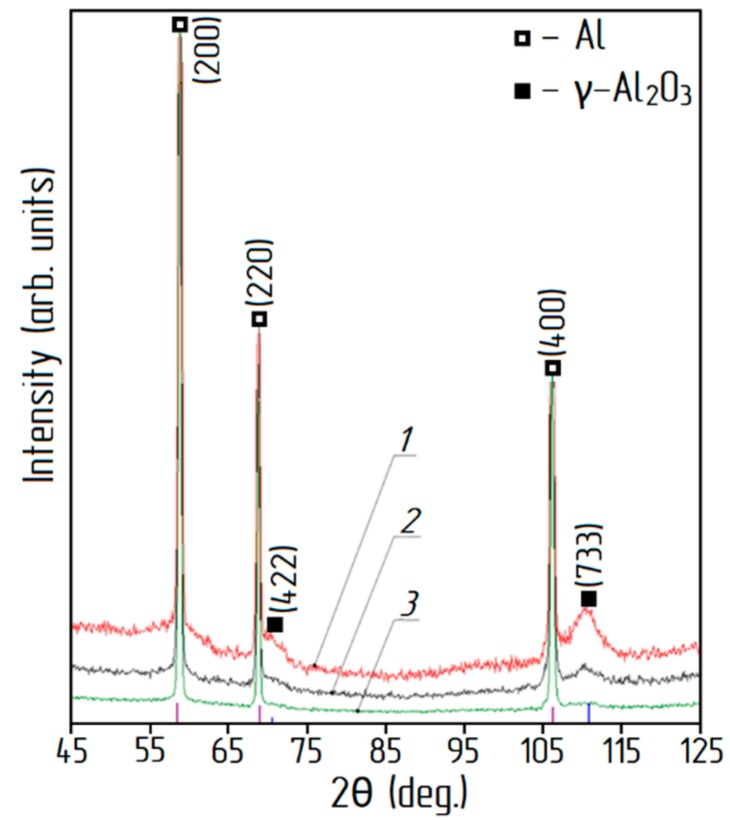
XRD patterns of aluminum-alumina powdery composites: 1) 20.0 wt. % Al_2_O_3_, 2) 14.5 wt. % Al_2_O_3_, 3) 10.0 wt. % Al_2_O_3_.

**Figure 8:**
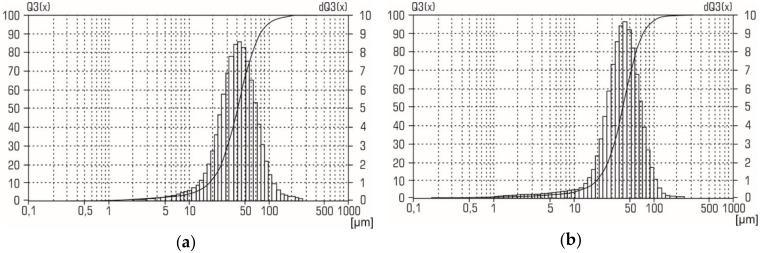
Particle size distribution of aluminum-alumina powder composites with the alumina content **a**) 10.0 wt. % and **b**) 20.0 wt. %.

XRD patterns for the aluminum-alumina powdery composites (after heating and calcination) for the different alumina contents are shown in Figure 7. Analysis of XRD patterns shows that in addition to aluminum, the presence of γ–Al_2_O_3_ was found. The relative intensity of the peaks corresponding to γ–Al_2_O_3_ correlates with the alumina content of the aluminum-alumina powdery composites. Absence of any other crystalline phase in the aluminum-alumina powdery composites was observed. Particle size distribution curves for aluminum-alumina powdery composites with alumina content of 10.0 wt. % and 20.0 wt. % are shown in Figure 8. Both powders (see Figure 8) have a rather narrow particle size distribution. More than 90% of the particles have a size from 20 to 80 µm. In general, the size distribution curves of aluminum-alumina powdery composites were the same as for the initial aluminum powders (see Figure 2).

The average particle size of aluminum-alumina composites was 41–42 µm. This value was also average for the initial aluminum powder (see Figure 2). The calculated value mean oxide film thickness for average particle varied from 1.5 µm (mode A) to 3 µm (mode D) (Figure 5). A small change in the particle size distribution curves was, probably, due to the swelling of the particles because of the lower density of the oxide layer compared to aluminum. This could be explained by the absence of pores in the obtained powders. This is a significant advantage in comparison with partially oxidized powers in gaseous (oxygen, carbon dioxide) mediums which usually have a large pore volume [24,38].

## 4. Conclusions

The method of obtaining aluminum-alumina powdery composites for their subsequent sintering by selective laser melting has been studied. Initial micron-sized aluminum powders were oxidized in water at 120–200 °C and 0.15–1.8 MPa pressure. Oxidation products were dried at 120 °C and calcined at 600 °C. Four high-pressure high-temperature oxidation modes were considered. The alumina content, particle morphology, and particle size distribution for the obtained aluminum-alumina powdery compositions were studied by XRD, SEM, laser diffraction, and volumetric methods.

The temperature of the oxidation process affected the alumina content in the composites. The alumina content was 10.0 wt. % for 120 °C and it was increased to 20.0 wt. % at 200 °C. The beginning of the oxidation reaction of aluminum for all modes was observed at 68 °C. 

The particles of the initial aluminum powder had a spherical shape and did not change significantly after processing. The average particle size and size distribution did not differ significantly from the initial aluminum powder indicating low porosity of the formed oxide layer. According to the obtained characteristics of aluminum-alumina powdery composites, they are suitable for further sintering. However, for the removal of agglomerates and additional sieving with 100 µm mesh size is necessary.

Synthesis of 3D objects from aluminum-alumina powdery composites by the SLM process will be considered in the future.

## Figures and Tables

**Table 1:** Parameters of obtaining aluminum-alumina powdery composites.

Mode	Parameters of Stage 1				
	Maximal Temperature, °C	Pressure, MPa	Volume of the Released H_2_, Liters	Alumina Content, wt. %	Mean Alumina Layer Thickness *, µm
A	120	0.15	65	10.0	1.45
B	180	1.35	190	14.5	2.14
C	190	1.50	260	17.4	2.60
D	200	1.80	300	20.0	3.00

*: Calculated for mean particle diameter particle.

**Table 2:** Parameters of obtaining aluminum-alumina powdery composites.

Element, wt. %	
Al	99.20
Ga	0.09
Zn	0.08
Ce	0.08
La	0.07
Fe	0.07
V	0.03
Mg	0.02
B	0.01
Cr, Ti, Co, Y, Cu, and volatiles	0.35
